# Vaccinations, Mobility and COVID-19 Transmission

**DOI:** 10.3390/ijerph19010097

**Published:** 2021-12-23

**Authors:** Jianfeng Guo, Chao Deng, Fu Gu

**Affiliations:** 1 Institute of Science and Development, Chinese Academy of Sciences, Beijing 100190, China; guojf@casipm.ac.cn (J.G.); dengchao191@mails.ucas.ac.cn (C.D.); 2 School of Public Policy and Management, University of Chinese Academy of Sciences, Beijing 100049, China; 3 Center of Engineering Management, Polytechnic Institute, Zhejiang University, Hangzhou 310027, China; 4 Department of Industrial and System Engineering, Zhejiang University, Hangzhou 310027, China; 5 National Institute of Innovation Management, Zhejiang University, Hangzhou 310027, China

**Keywords:** COVID-19, human mobility, non-pharmaceutical effectiveness, vaccination

## Abstract

In order to prevent the spread of coronavirus disease 2019 (COVID-19), 52.4% of the world population had received at least one dose of a vaccine at17 November 2021, but little is known about the non-pharmaceutical aspect of vaccination. Here we empirically examine the impact of vaccination on human behaviors and COVID-19 transmission via structural equation modeling. The results suggest that, from a non-pharmaceutical perspective, the effectiveness of COVID-19 vaccines is related to human behaviors, in this case, mobility; vaccination slows the spread of COVID-19 in the regions where vaccination is negatively related to mobility, but such an effect is not observed in the regions where vaccination and mobility have positive correlations. This article highlights the significance of mobility in realizing the effectiveness of COVID-19 vaccines; even with large-scale vaccination, non-pharmaceutical interventions, such as social distancing, are still required to contain the transmission of COVID-19.

## 1. Introduction

Vaccination is a primary pharmaceutical intervention against COVID-19, a pandemic that had already claimed 5,131,360 human lives globally at 19 November 2021 [1]. Examining the vaccine efficacy against COVID-19 is a task of critical importance, as intensive efforts have now been exerted to develop and inoculate the public with COVID-19 vaccines. According to Coronavirus Vaccine Tracker provided by the New York Times (updated on 16 November 2021) [2], there are 13 types of COVID-19 vaccines that have been administered, 132 vaccines are in clinical development, and 194 more vaccines are in pre-clinical development. On 16 November 2021, there are 4,132,325,886 people who have taken at least one dose of vaccine inoculation, and 3,251,575,096 people have received two-shot vaccination, making up 52.47% and 41.29% of the world’s total population, respectively [3].

However, compared to the extensive literature that examines the pharmaceutical efficacy of COVID-19 vaccines [4,5,6,7,8,9,10,11,12,13,14,15], the available knowledge on the non-pharmaceutical aspect of vaccination is extremely limited. Only related works focus on mathematically modeling the joint effect of vaccination and social distancing in containing the spread of COVID-19 [16,17,18,19,20], while there is no evidence about the non-pharmaceutical influence of vaccine inoculations. All of these modeling works suggest that the realization of the vaccine efficacy against COVID-19 is dependent on non-pharmaceutical interventions, like social distancing and face mask use [16,17,18,19,20], and such observation shows a good agreement with a cohort study [21]. This implies that the non-pharmaceutical aspect of vaccination could play a notable role in containing the transmission of COVID-19.

In this article, we empirically investigate the impact of vaccination on human behaviors, in this case, mobility, as well as on the transmission of COVID-19. Specifically, we try to answer one important research question: with altered human behaviors considered, are the COVID-19 vaccines still effective? To this end, we employ a structural equation modeling approach to examine the relationship of vaccination to mobility and to new COVID-19 cases. The two-step approach not only allows significance tests on the interactions of all parameters, but also enables assessments on the fitness and validity of any model of interest [22,23]. This method is substantially employed for causality analysis in the medical, social, and behavioral sciences [22,23,24,25,26,27,28].

**Figure 1:**
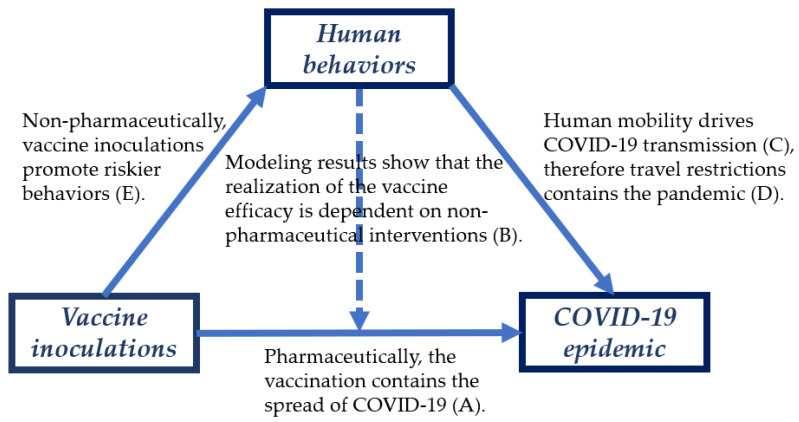
Interrelationships of vaccine inoculations, human mobility, and COVID-19 epidemic. In the figure, the dotted and solid line represent the observations of the modeling studies and empirical studies respectively. (**A**): [4,5,6,7,8,9,10,11,12,13,14,15]; (**B**): [16,17,18,19,20]; (**C**): [29,30]; (**D**): [30,31,32]; (**E**): [33,34].

In the structural equation modeling framework, human mobility is employed as a moderating variable. The selection is justified by the following facts. Firstly, mobility is one of the major drivers of COVID-19 transmission [29,30], and non-pharmaceutical interventions, like travel restrictions, are effective in containing this pandemic [30,31,32]. The related modeling works suggest that such measures should not be prematurely removed during vaccination, otherwise the spread of COVID-19 will still be accelerated [17,18,19,20]. Secondly, vaccination could have an impact on human behaviors; extensive evidence suggests that vaccine inoculations could lower the risk perception of vaccinators, and thereby promote riskier behaviors [33,34]. The logic of this research is shown as Figure 1.

In detail, our article offers three major contributions. First, we pioneer to empirically examine the non-pharmaceutical influence of COVID-19 vaccine inoculations on the containment of the pandemic. Despite the pharmaceutical efficacy of COVID-19 vaccines, which has been intensively investigated [4,5,6,7,8,9,10,11,12,13,14,15], currently there is no available knowledge about the vaccines’ non-pharmaceutical efficacy. Second, our work is amongst the first to consider the impact of vaccination on human mobility, which is a primary driver of the epidemic spread and can potentially be influenced by risk perception. Third, we identify the differentiated causal relations between vaccine inoculations and reported COVID-19 cases in different groups of countries, offering empirical evidence to support the formulation of pandemic control measures; a delicate and challenging task in the era [20].

## 2. Materials and Methods

### 2.1. Variables and Sources

In this article, we acquire the data of newly reported COVID-19 cases (denoted as new_cases) and accumulative vaccine inculcations (denoted as total_vaccinations) from Our World in Data [3], a program of Global Change Data Lab that gathers COVID-19-related official figures from governments and health ministries. Here we select the data of total vaccinations, which refer to the accumulative figure of the COVID-19 vaccine inoculations in one country up to a specific date.

The daily mobility data at the country-level during the COVID-19 pandemic is collected from Community Mobility Reports provided by Google [35]. These reports are created with aggregated, anonymized sets of data from the Google users on mobile devices who have turned on the Location History setting worldwide. The mobility data is in baseline form, that is, the mobility for the report date is compared to that of the baseline. In this work, the median value of the mobility figures reported between 3 January 2020 to 6 February 2020 is employed as the baseline. The mobility data is gathered according to the same standard with unqualified data filtered out; therefore, this dataset can support our research objective despite possible measurement errors.

COVID-19 vaccine inoculation started in December 2020 [3], six months before and after the beginning of inoculation is selected as our sample; that is, our studied period spans from 6 June 2020 to 6 June 2021. Since several countries, such as Australia [36] and New Zealand [37], have not lifted lockdown or issued travel restrictions after the starting of vaccination, the interactions of vaccination, mobility and reported cases could show some degree of regional heterogeneity. Thus, a set of daily panel data that consists of 124 countries is obtained, spanning from 6 June 2020 to 6 June 2021.

### 2.2. Country Classification

Prior to the main empirical estimation, we employ multiple regression analysis to determine the valuence of country-level vaccination-mobility relations and mobility-cases relations, which are based on Formulas (1) and (2) respectively.

(1) mobility=a0+a1×total_vaccinations(−28)+x1+x2+x3+x4+x5+x6

where xi denotes the ith day of a week; for instance, x1 denotes Monday, x2 denotes Tuesday, and ai are the coefficients of variables. The lag period of 28 days is commonly adopted in the inoculations of COVID-19 vaccines, such as the Pfizer-BioNTech COVID-19 Vaccine [38].

(2) new_cases=a0+a1×mobility(−7)+x1+x2+x3+x4+x5+x6

where the seven-day incubation period is derived from the epidemic literature [39].

**Figure 2:**
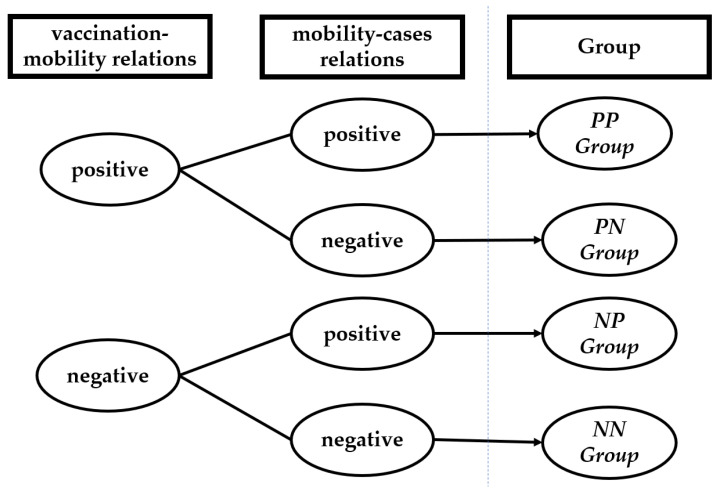
Classification criteria for the 124 included countries. Based on vaccination-mobility relations and mobility-cases relations, four groups, namely PP group, PN group, NP group, and NN group, are identified.

**Figure 3:**
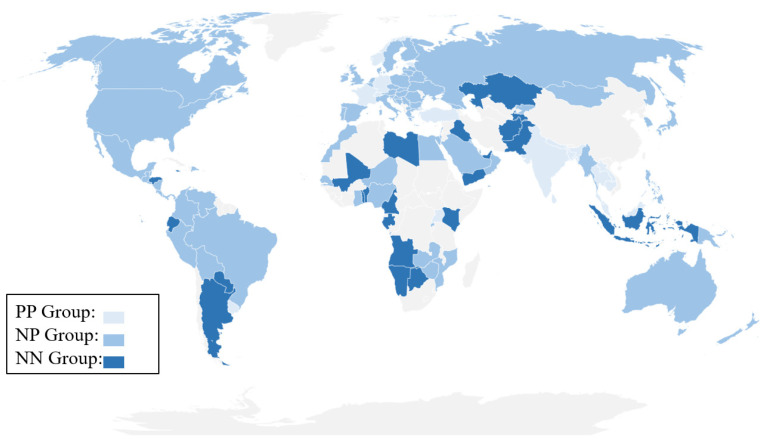
The categorization of the 124 included countries. The countries belong to the PP group are marked in light blue, the countries belong to the NP group are marked in blue, and the countries belong to the NN group are marked in dark blue.

According to the valuence of the vaccination–mobility relations and mobility–cases relations (identified by correlation analysis) at the country level, we classify the 124 included countries into four categories, namely, the countries that have positive vaccination–mobility relations and positive mobility–cases relations (denoted as PP group), the countries that have positive vaccination–mobility relations and negative mobility–cases relations (denoted as PN group), the countries that have negative vaccination–mobility relations and positive mobility–cases relations (denoted as NP group), and the countries that have negative vaccination–mobility relations and negative mobility–cases relations (denoted as NN group). The classification procedure is displayed in Figure 2, and the categories of the 124 included countries are shown in Figure 3. The complete list is shown in Table A1 in Appendix A. The PN group is omitted in the following calculation, as it only contains three countries, see Table A1.

### 2.3. Structural Equation Modeling

Our structural equation modeling approach is comprised of the three models. The structural equation model helps to verify the relationship between variables, though the approach cannot directly determine causality. The first model (Model I) examines the relation of the accumulative vaccine inoculations to the numbers of newly reported COVID-19 cases, specified as follows:

(3) new_cases=a0+a1×total_vaccinations(−28)+x1+x2+x3+x4+x5+x6

The second model (Model II) examines the relation of the human mobility to the numbers of newly reported COVID-19 cases, specified as follows:

(4) new_cases=a0+a1×mobility(−7)+x1+x2+x3+x4+x5+x6

The third model (Model III) examines the relation of the accumulative vaccine inoculations to the numbers of newly reported COVID-19 cases, with the human mobility as a moderating variable, specified as follows:

(5) new_cases=a0+a1×total_vaccinations(−28)+a2×mobility(−7)+a3×moderater+x1+x2+x3+x4+x5+x6

where the other moderator is defined as the product of the centered values of total_vaccinations and mobility, shown as follows:

(6) $$\mathrm{moderater}=v_{\mathrm{center}}\left(-28\right)\times r\_\mathrm{center}\left(-7\right)$$

## 3. Empirical Results

### 3.1. Results of Structural Equation Modeling

Table 1 displays the relations of vaccination to COVID-19 cases in the PP group, NP group, NN group and at the global level, with and without considering mobility.

From Table 1 (a), without considering the moderating effect of mobility, inoculation of COVID-19 vaccines shows a positive correlation with the numbers of newly reported COVID-19 cases in the PP group; the coefficient of total_vaccinations is 0.0016 (*p* = 0.000), which indicates that every additional 1000 vaccine inoculations could lead to 1.559 additional cases. With the inclusion of mobility, the relation of COVID-19 vaccine inoculations to the cases is strengthened; the coefficient of total_vaccinations is 0.0023 (*p* = 0.000), which means that every additional 1000 vaccine inoculations are related to 2.275 additional cases, being 46.23% higher than the value that excludes mobility. In addition, mobility promotes the spread of the pandemic, as its coefficient is 439.743.

As shown in Table 1 (b), vaccine inoculations are helpful in containing the transmission of COVID-19 in the NP group; the coefficient of total_vaccinations is −0.0003 (*p* = 0.000), implying that every additional 1000 vaccine inoculations could result in a reduction of 3.183 cases. Considering the impact of mobility, the ameliorating effect of vaccines are enhanced, as every additional 1000 vaccine inoculations could further reduce 4.002 cases. In the NP group, one additional unit of mobility could be responsible for 119.676 COVID-19 cases.

Table 1 (c) shows that, in the absence of mobility, vaccination could propel the spread of COVID-19 in the NN group; every additional 1000 vaccine inoculations could trigger 0.301 additional COVID-19 cases. The propelling effect is enhanced with the consideration of the moderating variable, i.e., mobility, which has a negative relation to COVID-19 transmission in this group. With mobility included, every additional 1000 vaccine inoculations could be related to 0.3598 additional COVID-19 cases.

### 3.2. Results of Granger Causality Analysis

**Figure 4:**
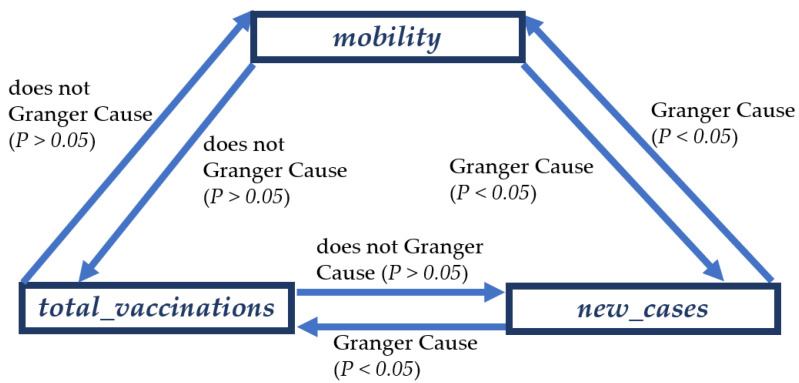
The significance of the results of the Granger causality tests on the three variables, i.e., the newly reported COVID-19 cases, accumulative vaccine inculcations, and human mobility in the NN group. Amongst the six tested relations, the human mobility is a Granger cause of the newly reported COVID-19 cases, which are one Granger cause of the accumulative vaccine inculcations. However, the other relations fail the Granger test, as they are not sufficiently significant.

Further, we test the Granger causality among the three variables in the NN group, and we find that the new reported COVID-19 cases are the Granger causes of vaccination and mobility (see Figure 4). The propelling effect of the pandemic on vaccine inoculations can be attributed to increased risk perceptions and self-preservation behaviors, which agrees with the outcomes of the existing surveys on the perception of the pandemic and vaccine intensions [40,41,42]. The Granger causal relation between COVID-19 cases and mobility might be explained by increased vaccine inoculations; with reported cases increased, people are rushing to vaccination sites to get inoculated. It is worth noting that the Granger causality test results indicate data causality, not fact causality.

Globally, the relations of vaccination to mobility and mobility to newly reported COVID-19 cases are both positive, being similar to those of the PP group. Therefore, the observed pattern in Table 1 (d) is also like that of the PP group. At the global level, the inoculations of COVID-19 vaccines are significantly and positively related to the spread of this pandemic; every additional 1000 vaccine inoculations are correlated to 0.164 additional COVID-19 cases. The relation almost remains unchanged with the consideration of mobility, as every additional 1000 inoculations still lead to 0.164 additional cases. Our structural equation modeling also shows that mobility exhibits a positive impact on the pandemic’s transmission; one additional unit of mobility is linked to 154.074 additional COVID-19 cases. Again, human mobility is confirmed to be a major driver of the spread of COVID-19.

## 4. Discussion

Vaccination is regarded as containing the spread of COVID-19; a pandemic that has been rampant around the globe since 2020. However, such faith has no empirical backups, although the pharmaceutical efficacy of COVID-19 vaccines has been intensively investigated [4,5,6,7,8,9,10,11,12,13,14,15]. Here we seek to verify the non-pharmaceutical effectiveness of vaccine inoculations, as vaccination could interact with non-pharmaceutical interventions [16,17,18,19,20] and alter human behaviors [33,34]. Based on a structural equation modeling approach, our empirical estimation shows that vaccination might facilitate the transmission of COVID-19, and mobility enhances such a positive relation. In other words, from a non-pharmaceutical perspective, the vaccination against the spread of COVID-19 is not always effective when human behaviors are altered, providing empirical explanation to the observed differences in the outcomes of COVID-19 vaccination [43].

We find that at the global level, the progress of vaccination is positively correlated to human mobility, which drives the transmission of COVID-19; with altered human behaviors considered, the inoculations of COVID-19 vaccines are positively related to the spread of the pandemic. Our results suggest that similar patterns are found in the countries that have positive vaccination–mobility relations and positive mobility–cases relations (the PP group) and the countries that have negative vaccination–mobility relations and negative mobility–cases relations (the NN group), while the vaccine inoculations reduce the COVID-19 transmission in the countries that have negative vaccination–mobility and positive mobility–cases relations (the NP group). Furthermore, we show that the reported COVID-19 cases are Granger causal causes of vaccine inoculations in the NN group. Our observation explains the rationality of the negative vaccination–mobility and mobility–cases relations, as the perceived risk of this pandemic is a major driver of self-protective behaviors and vaccine acceptance [40,41,42,44].

The observed differentiated relations between vaccine inoculations and new cases suggest that human behaviors (in this case, mobility) would have an impact on the outcomes of COVID-19 vaccines. The findings offer empirical evidence to support the results of the theoretical modeling works [16,17,18,19,20]; the premature removal of travel restrictions could facilitate the spread of the pandemic, and thereby compromise the overall effectiveness of vaccination. Neither the general public nor authorities can put the hope of controlling this epidemic entirely on vaccination.

Admittedly, our work suffers from three major limitations. First, we perform the estimation based on macro-level data, as there is no available individual-level data of vaccine inoculation, COVID-19 infection, and travel behaviors. Second, it is highly likely that different types of COVID-19 vaccines (e.g., genetic vaccines like mRNA-1273 developed by Moderna and Comirnaty developed by Pfizer, viral vector vaccines like Ad26.COV2.S developed by Johnson & Johnson(One Johnson & Johnson Plaza, New Brunswick, NJ 08933, United States), protein-based vaccines like NVX-CoV2373 developed by Novavax, and inactivated or attenuated vaccines like CoronaVac developed by Sinovac Biotech(Peking University Biological City, No. 39 Shangdi West Road, Haidian District, Beijing 100085, China.) and VLA2001 developed by Dynavax(2100 Powell Street, Suite 900, Emeryville, CA 94608, United States)) might have different effects in providing immunity against COVID-19, but they are not clearly distinguished. Third, the last limitation lies in the human mobility data employed in this work. Google’s Community Mobility Reports are aggregated from application users who have turned on the Location History setting, the default setting of which is off. Therefore, the representativeness of the mobility data is constrained.

## 5. Conclusions

Vaccination is considered as a primary measure to contain the spread of COVID-19. Though the pharmaceutical effectiveness of COVID-19 vaccines has been intensively investigated, there is little knowledge about the non-pharmaceutical aspect of vaccine inoculations that is currently available. Using a structural equation modeling framework, here we empirically examine the relation of vaccination on human mobility, as well as to COVID-19 transmission. We observe that the non-pharmaceutical effectiveness of vaccination is related to its relation to mobility, as vaccination is non-pharmaceutically effective only in the countries where vaccination has a negative impact on mobility. Our findings stress the importance of non-pharmaceutical interventions (e.g., travel restrictions) against COVID-19, as premature removal of such measures could significantly compromise the effectiveness of vaccines.

## Figures and Tables

**Table 1:** The relations of the COVID vaccine inoculations to the numbers of newly reported COVID-19 cases.

Group	Model	Total_Vaccinations	Mobility	Moderator
(a) PP Group	I	0.0016 *** (*p* = 0.000)		
	II		439.7427 *** (*p* = 0.000)	
	III	0.0023 *** (*p* = 0.000)	79.1345 *** (*p* = 0.000)	−0.0001 *** (*p* = 0.000)
(b) PN Group	I	−0.0003 *** (*p* = 0.000)		
	II		119.6760 *** (*p* = 0.000)	
	III	−0.0004 *** (*p* = 0.000)	75.1117 *** (*p* = 0.000)	−0.0001 *** (*p* = 0.000)
(c) NN Group	I	0.0003 *** (*p* = 0.000)		
	II		−25.5850 *** (*p* = 0.000)	
	III	0.0004 *** (*p* = 0.000)	−15.1933 *** (*p* = 0.000)	0.0001 *** (*p* = 0.000)
(d) Global Level	I	0.0002 *** (*p* = 0.000)		
	II		154.0744 *** (*p* = 0.000)	
	III	0.0002 *** (*p* = 0.000)	165.3865 *** (*p* = 0.000)	0.000056 *** (*p* = 0.000)

The results of Model I, II, and III of (a) the PP group, (b) the NP group, (c) the NN group, and (d) the global level. *** *p* < 0.001 based on a chi-squared test.

## Data Availability

Data are available in a publicly accessible repository.

## Appendix A

**Table A1:** Categorized countries that are included in this study.

Category	Countries	Quantity
PP group	Bangladesh, Cambodia, Estonia, Fiji, France, Germany, India, Laos, Malaysia, Mauritius, Nepal, Norway, Singapore, Sri Lanka, Switzerland, Thailand, Trinidad and Tobago, Turkey, Uganda, Uruguay	20
PN group	Chile, Jordan, Vietnam	3
NP group	Australia, Austria, Barbados, Belarus, Belgium, Belize, Bolivia, Bosnia and Herzegovina, Brazil, Bulgaria, Canada, Colombia, Costa Rica, Croatia, Czechia, Denmark, Dominican Republic, Egypt, El Salvador, Finland, Georgia, Ghana, Greece, Guatemala, Hungary, Ireland, Israel, Italy, Japan, Kuwait, Kyrgyzstan, Latvia, Lebanon, Lithuania, Luxembourg, Malta, Mexico, Mongolia, Morocco, Mozambique, Myanmar, Netherlands, New Zealand, Nicaragua, Niger, Nigeria, North Macedonia, Oman, Panama, Papua New Guinea, Peru, Philippines, Poland, Portugal, Qatar, Romania, Russia, Rwanda, Saudi Arabia, Senegal, Serbia, Slovakia, Slovenia, South Korea, Spain, Sweden, Ukraine, United Kingdom, United States, Venezuela, Zambia, Zimbabwe	72
NN group	Afghanistan, Angola, Antigua and Barbuda, Argentina, Bahrain, Benin, Botswana, Cameroon, Cape Verde, Ecuador, Gabon, Honduras, Indonesia, Iraq, Jamaica, Kazakhstan, Kenya, Libya, Mali, Namibia, Pakistan, Paraguay, Tajikistan, Togo, United Arab Emirates, Yemen	26
